# Multifocal oral melanoacanthoma associated with Addison’s disease and hyperthyroidism: a case report

**DOI:** 10.1590/2359-3997000000273

**Published:** 2017-06-14

**Authors:** Thinali Sousa Dantas, Isabelly Vidal do Nascimento, Maria Elisa Quezado Lima Verde, Ana Paula Negreiros Nunes Alves, Fabrício Bitu Sousa, Mário Rogério Lima Mota

**Affiliations:** 1 Universidade Federal do Ceará Fortaleza CE Brasil Divisão de Patologia Oral, Universidade Federal do Ceará (UFC), Fortaleza, CE, Brasil; 2 Universidade Federal do Ceará Fortaleza CE Brasil Universidade Federal do Ceará (UFC), Fortaleza, CE, Brasil

## Abstract

Oral melanoacanthoma is a mucocutaneous, pigmented, rare, benign, and probably reactive lesion. This paper reports for the first time in the literature a case of multifocal oral melanoacanthoma in a patient diagnosed with Addison’s disease and concomitant Graves’ disease with hyperthyroidism. The patient presented with oral pigmented lesions, which were hypothesized to be mucosal pigmentation associated with Addison’s disease. Due to their unusual clinical pattern, these oral lesions were biopsied and diagnosed as oral melanoacanthoma on histopathology and immunohistochemistry for HMB-45. At the moment of this report, the patient was being treated for her systemic conditions, but the lesions had not regressed. Reactive hyperpigmentation of the skin and mucous membranes may be found in Addison’s disease and hyperthyroidism. This case reinforces the hypothesis of a reactive nature for oral melanoacanthoma and highlights the need for investigation of endocrine disorders in patients with multifocal oral melanoacanthoma.

## INTRODUCTION

Cutaneous melanoacanthoma was first described as a benign tumor with characteristics of a pigmented variant of seborrheic keratosis, presenting as a proliferation of keratinocytes and dendritic melanocytes in the lower portion of the epithelium (1). However, melanoacanthoma affecting the oral cavity seems to have a more reactive than truly neoplastic characteristic, including a flat or slightly elevated surface, fast growth, preference for involving mucosa exposed to trauma, partial or complete regression after removal of the irritant, as well as association with inflammatory processes. Based on that, the term oral melanoacanthoma has been used to characterize these particular lesions (2-4).

The oral manifestation may present as a single or, less frequently, multiple lesions affecting almost exclusively dark-skinned patients with a predilection for poor young females (5-8).

Oral melanoacanthoma lesions may vary in color from dark brown to black and are often asymptomatic, although in some cases pain, itching, and burning may occur (5,9). The buccal mucosa is the most affected site, but the lesions may also be found on the palate, gingiva, and lip mucosa (5,7,10).

Histologically, oral melanoacanthoma is characterized by a proliferation of benign dendritic melanocytes across the epithelium, spongiosis, acanthosis, chronic inflammatory infiltrates, and eosinophils in the underlying connective tissue (9,11).

The diagnosis of oral melanoacanthoma is based on the histological assessment of the lesion. The Fontana-Masson stain and an immunohistochemical profile for melanocytes, such as the S-100 protein, Melan-A, and HMB-45, can be used to verify the presence of melanin (7,8,10).

Several melanocytic lesions can sometimes resemble oral melanoacanthoma, including melanotic macule, melanocytic nevus, atypical melanocytic proliferation/hyperplasia, and melanoma; (3) therefore, biopsy is indicated to establish the diagnosis (6,10,11). Until the biopsy report becomes available, no further treatment is necessary since the lesion may undergo spontaneous regression, although rarely it may relapse and additional lesions may appear (8,10).

Multiple pigmented lesions of the oral cavity may present clinical features of several systemic disorders such as Addison’s disease, Peutz-Jeghers syndrome, and, to a lesser extent, hyperthyroidism (8,10,12,13).

Addison’s disease is an endocrine disorder characterized by a deficient production of adrenal cortex hormones leading to increased secretion of adrenocorticotropic hormone (ACTH) (8,10,14). The most common symptoms of Addison’s disease are fatigue, loss of appetite and weight, weakness, and hypotension. In addition to these symptoms, patients with Addison’s disease also have generalized hyperpigmentation of the skin and oral mucosa (8,10,14).

The oral lesions usually precede the cutaneous manifestations, which can be an important diagnostic feature in the early stages of the disease. These lesions affect mainly the lips, buccal mucosa, gingiva, palate, and tongue (8,10,12,13). Treatment of these lesions is not required since they disappear during treatment of the disease itself (8,10).

Hyperthyroidism is also an endocrine disorder, in which excessive thyroid hormone is synthesized and released. This disorder may be associated with several etiologies, such as cancer and autoimmune diseases (Graves’ disease) (15). Cutaneous manifestations of hyperthyroidism may appear in the form of hyperhidrosis, myxedema, eczematous dermatitis, alopecia, hyperpigmentation, and telangiectasia. These pigmented lesions affect approximately 2% of the patients with hyperthyroidism. They are poorly described in the literature and rarely involve mucous membranes (13,16).

This article aims to describe for the first time in the literature a case of multifocal oral melanoacanthoma in a patient with concomitant Addison’s and Graves’ disease.

## CASE REPORT

A 50-year-old dark-skinned woman presented to the Stomatology Service of the Federal University of Ceará, Brazil, complaining of two dark patches on her upper lip mucosa. During anamnesis, the patient reported that these patches had grown in recent months, accompanied by a slight itching in the area. She also reported having both Addison’s and Graves’ disease. Information retrieved from her medical history showed that the diagnosis of Graves’ disease with hyperthyroidism was established in 2014 based on clinical characteristics presented on thyroid scintigraphy and blood tests (thyroid-stimulating hormone [TSH] 0.01 mU/L) and anti-TSH receptor antibody [TRAB] 2.68 IU/L). A current serum free thyroxine (FT4) measurement was 2.04 ng/dL. The adrenal insufficiency was diagnosed in the same period that the pigmented lesions had appeared, with a serum cortisol level of 2.3 µg/dL and ACTH level of 84.5 ng/L. At the time of this report, the patient was being treated and monitored for both these diseases.

**Figure 1:**
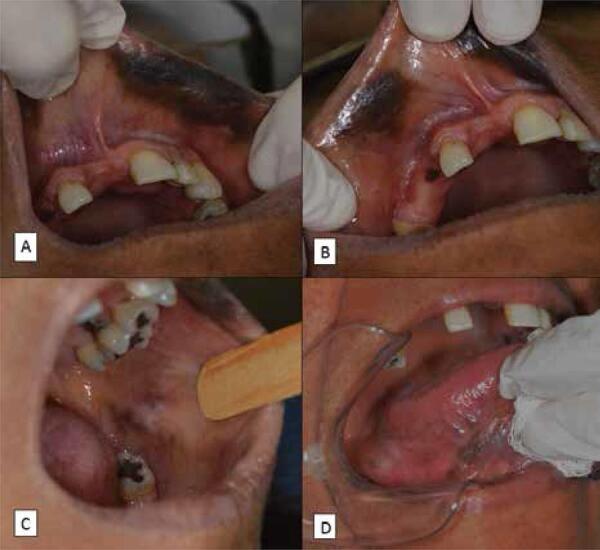
Images showing the presence of brown and black patches and plaques distributed in the (A) upper lip mucosa, (B) gingiva, (C) buccal mucosa, and (D) tongue.

On clinical examination, she presented brown and black patches and plaques sometimes permeated by whitish areas. These lesions were asymptomatic, had varying sizes with irregular and imprecise limits, and were distributed in the upper lip mucosa, gingiva, tongue, and buccal mucosa (Figure 1). The patient used a dental prosthesis, but no trauma from the prosthesis was observed in association with the pigmentation. During the physical examination, these lesions were not observed on the skin. Due to the multifocal involvement and the presence of associated systemic diseases, the hypothesis of a mucosal pigmentation associated with Addison’s disease was proposed. However, due to lack of skin pigmentation, distinct clinical appearance, and history of increased lesion size in recent months, a biopsy of the lesions in the upper lip mucosa was performed.

**Figure 2:**
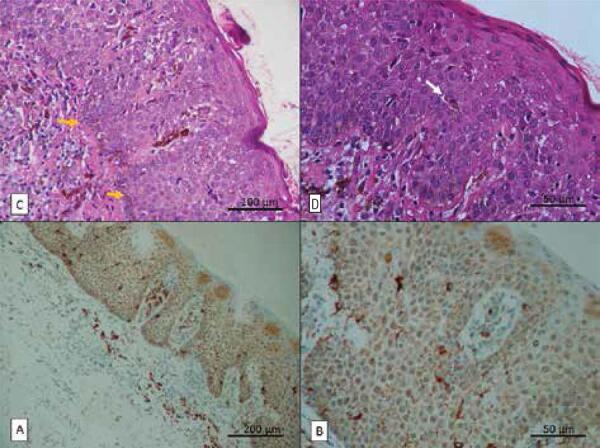
Oral melanoacanthoma. (A) Stratified squamous epithelium with hyperparakeratinized areas showing acanthosis, substantial deposition of melanin in basal and suprabasal layers (yellow arrows); (B) dendritic melanocytes (white arrow) in all epithelial layers (hematoxylin-eosin [H&E] stain, original magnification 200X and 400X, respectively); (C) and (D) positive HMB-45 expression in dendritic melanocytes extending across the epithelium (immunohistochemical reaction, original magnification 100X and 400X, respectively).

The histopathological report described a stratified squamous epithelium with hyperparakeratinized areas showing acanthosis, spongiosis, exocytosis, vacuolar degeneration, substantial deposition of melanin in all epithelial layers, and a suggestive melanocytic hyperplasia, with occasional dendritic melanocytes in all epithelial layers. The adjacent connective tissue presented subepithelial melanophages and mononuclear cell infiltration (Figure 2 A and B), consistent with oral melanoacanthoma. Immunohistochemistry for HMB-45 showed dendritic melanocytes extending across the epithelium, confirming the diagnosis of oral melanoacanthoma (Figure 2 C and D).

At the time of this report, the patient was undergoing treatment with follow-up assessments every 3 months and using Tapazol 5 mg for hyperthyroidism. No medication for Addison’s disease had been initiated, and the cortisol levels were within the normal range (TSH 1.33 mU/L, FT4 0.87 ng/dL, cortisol 13.59 µg/dL). No changes in the clinical appearance of the pigmented lesions were observed during follow-up.

## DISCUSSION

Addison’s disease comprises all etiologic factors destroying the adrenal cortex. (17) Patients with this disease may have other autoimmune disorders, classified as autoimmune polyglandular syndromes (17). In addition to Addison’s disease, the patient in the present report also had Graves’ disease, characterized by the presence of autoantibodies binding to the TSH receptor, stimulating the growth of the thyroid gland and secretion of thyroid hormones. Graves’ disease is one of the main causes of hyperthyroidism (18).

Reactive mucosal hyperpigmentation may be a common finding in Addison’s disease, since cortisol deficiency leads to increased production of ACTH, a pituitary hormone with a chemical structure similar to that of melanocyte-stimulating hormone (MSH), stimulating the pigmentation of the skin and mucosa (14,19). Hyperthyroidism may also be associated with hyperpigmentation, but pigmented lesions are rarely described in the literature and generally do not involve the mucosa, with only about 2% of the patients having associated skin pigmentation (13,16).

Histopathological examination of the pigmented lesions associated with Addison’s disease shows acanthosis and heavy deposition of melanin in the basal and subepithelial layers (20). Pigmented lesions associated with hyperthyroidism share a similar histopathology to those of Addison’s disease, but with a clear deposition of hemosiderin in the connective tissue (13,20). There have been no reports in the literature of multifocal oral melanoacanthoma in a patient with concomitant Addison’s and Graves’ disease with hyperthyroidism.

The first description of oral melanoacanthoma was as a single lesion, reported by Schneider in 1981, but reports of multiple lesions are available in the literature (9,21). These lesions are slightly elevated, brownish/blackish, usually affecting female and melanodermic patients, and requiring no treatment, as in the patient described in this report. The differential diagnosis of these lesions include nevi and melanomas, and a biopsy is required to confirm their diagnoses (6).

Unlike skin melanoacanthoma, oral melanoacanthoma appears to have a reactive nature, (2-4) and the case described here reinforce this theory. Increased ACTH levels can induce melanocyte proliferation and melanin secretion (19). In our patient’s case, her ACTH level was 84.5 ng/L. In addition, a possible association between thyroid hormones and their stimulating action on melanocytes has also been described (22,23). These hormonal effects may enhance the proliferation and activation of melanocytes causing melanocytic hyperplasia and deposition of melanin, as seen in the histopathology of oral melanoacanthoma. Besides, several experimental and *in vitro* studies demonstrate the role of thyroid hormones on epithelial proliferation (24,25), which could be related to the acanthosis observed in oral melanoacanthoma. This case suggests that hormonal changes may facilitate or amplify the emergence of oral melanoacanthoma in patients predisposed to this type of lesion.

In 2007, Carlos-Bregni and cols. (6) suggested a link between oral melanotic macule (reactive lesion with prominent basal melanosis) and oral melanoacanthoma. These authors reported that the activation of melanocytes by unknown mechanisms might be the link between these two lesions. Hormonal mechanisms may mediate this process.

HMB-45 is an antibody against the GP100 protein, which is present in melanosomes and, more commonly, in proliferating cells. This antibody is an important marker used in the diagnosis of melanomas with cytoplasmic positivity. It has a diagnostic role in helping differentiate melanomas and non-malignant lesions (11,26). It also reacts promptly with melanocytes of oral intramucosal nevi, including blue nevi (11). In our analysis, we found immunoreactivity for HMB-45 in melanocytes without atypia suggestive of malignancy, dendritic and non-dendritic, and in all epithelial layers.

Treatment in cases of oral melanoacanthoma is not recommended. However, these lesions must be followed up and may sometimes spontaneously disappear or decrease in size (3,9,27). The lesions in the present case remained unchanged during follow-up; however, changes in the clinical pattern of the lesions and their prognosis were still unavailable due to the short follow-up time and absence of similar cases in the literature. Finally, this report highlights the importance of investigating endocrine disorders (Addison’s and/or Graves’ disease) in patients diagnosed with multifocal oral melanoacanthoma.
